# Indications and appropriateness of caesarean sections performed in a tertiary referral centre in Uganda: a retrospective descriptive study

**DOI:** 10.11604/pamj.2017.26.64.9555

**Published:** 2017-02-03

**Authors:** Jonathan Peter Nelson

**Affiliations:** 1 Fort Portal Regional Referral Hospital, Uganda

**Keywords:** Caesarean section, global health, maternal health, medical education

## Abstract

**Introduction:**

The WHO has identified an ideal caesarean section rate for a nation of 10-15%, but much higher rates are seen in tertiary referral centres in resource-poor countries. Interventions by the author to improve care and reduced unnecessary caesareans were undertaken including staff education and production of clinical guidelines. This study aimed to identify indications for caesareans and whether the decision to perform caesareans was appropriate in order to improve care, and whether the above interventions had an impact on this process.

**Methods:**

Two groups of 100 consecutive cases from October 2014 and 100 from February 2015 were retrospectively selected that resulted in caesarean. These case notes were analysed for demographic data, caesarean indication and appropriateness.

**Results:**

In 46% of cases the decision for caesarean was considered appropriate. No significant difference (p>0.05) was found between the two groups in terms of patient demographics or appropriateness of caesarean (43% in Oct-14 compared to 48% in Feb-15). The most common group of indications for caesarean was dystocia (43.5%) with 28% appropriate; followed by fetal distress (18.5%) with 30% appropriate; previous scar (17%) with 85% appropriate; malpresentation (10.5%) with 48% appropriate; and maternal compromise (10%) with 80% appropriate.

**Conclusion:**

The high number of unnecessary caesareans appeared to be related to lack of knowledge and inexperience of staff. Despite attempts to address this through teaching the scope of the problem is so large it needs a fundamental change in the healthcare system in terms of resources, education, continuing professional development and clinical governance.

## Introduction

The World Health Organization (WHO) has identified an ideal caesarean section (CS) rate for a nation of around 10-15% [1, 2]. This is based on studies that show improving maternal and neonatal morbidity and mortality as rates rise up to this level, but minimal improvements or even negative health outcomes as rates increase past 10% [3, 4]. However, it is very difficult for an individual unit or hospital to use this information as an audit tool or a comparison due to the number of complex issues that impact on CS rates. In the UK national bodies have set a target caesarean rate of <23% [5], but in this resource-rich national health-care setting there are less factors that might cause large variations in rates between different hospitals. In resource-poor countries like Uganda, there is often a model of care where each region will have a tertiary centre where complex cases and severely obstructed labours might be referred, small units that perform normal deliveries, and large rural regions with traditional birth attendants. To know what an ideal CS rate for a unit like this is difficult. A number of studies from tertiary referral centres in Nigeria report CS rates of 11.3 – 35.5% [6–8]. The rate in our unit where this study was based was 32% in 2014 [9]. Whilst these rates are generally well above the WHO recommendation, there are limited studies exploring whether these higher rates are acceptable in the context of the caseload. The objectives of this study were to look at the indications for CS, the appropriateness of this decision for CS and what alternative management might have been offered in order to explore why the CS rate was at this level. In addition, educational interventions were instigated that might be considered a normal part of clinical governance and quality improvement, to see if these might improve the appropriateness of the decision for CS.

## Methods

Fort Portal Regional Referral Hospital is a government tertiary hospital serving the town of Fort Port and the surrounding region of Kabarole in Uganda. It receives referrals of cases from other hospitals and health centres in the region. There are approximately 7000 deliveries per year in the unit. The unit has 3 seniors’ doctors and 4-5 intern doctors working in obstetrics and gynaecology, alongside anaesthetic officers, students, midwives and nursing staff. Resources are limited in keeping with a government hospital in a resource-poor setting, and fetal monitoring is in the form of intermittent auscultation. This was a retrospective descriptive study of 200 cases that ended in caesarean section between October 2014 and February 2015. The first 100 cases were consecutive caesareans from October 2014 identified when discharged from the hospital. The case notes were used to collect socio-demographic data and other relevant clinical information on a self-designed proforma. This information included the primary indication for CS, whether the decision for CS was appropriate according to national guidelines, and who made the decision for CS. After this the author started working at the unit as a volunteer obstetrician, and as an additional senior doctor, supporting the intern doctors and midwifery/nursing staff. Various educational initiatives were implemented with the broad intention of improving care, particularly intra-partum care and the decision for caesarean section. These interventions included communication of results from the initial audit of 100 cases; regular bedside teaching sessions and ward rounds; continuing medical education sessions; and development of local guidelines (which in terms of decision for CS were similar to the national guidelines used to analyse the first 100 cases) surrounding intra-partum care and caesarean section. In February 2015 a further 119 consecutive caesarean section cases were identified, and 19 excluded due to direct involvement by the author, leaving 100 cases to analyse using the same proforma as previous. Appropriate statistical tests were applied when comparing the demographic data between the two groups (e.g. October 2014 and February 2015) using Graph Pad Scientific Software. Depending on the data set this was an unpaired t-test, or a two-way Fisher’s exact test. The level set for statistical significance was p < 0.05. No power calculations were performed as the original data was collected in the context of an audit rather than interventional study. Permission for the original audit work was given by the hospital director and head of department and it was presented locally. Advice was sought from a national ethics board about the need for further approval to use the data in the context of this analysis and it was not felt to be necessary. Data was anonymised appropriately.

## Results

Of the 200 cases the mean age was 23.9 years with a range of 15-43 years. There were 78 primiparous women (39%). 129 women had no previous caesarean sections (64.5%), 38 one previous caesarean section (19%) and 33 two or more previous caesarean sections (16.5%). The median gestation for delivery was 39 weeks, with a range of 28-43 weeks gestation. There was no difference in demographic data between the 2 groups from October 2014 and February 2015 (p>0.05) (Table 1). The indications recorded for CS were put into 5 main groups consistent with other literature in the field [6, 10]. The most common indication was dystocia in 44% (n=88), followed by presumed fetal distress in 18.5% (n=37), high risk of uterine rupture in 17% (n=34), malpresentation in 10.5% (n=21) and maternal/fetal compromise 10% (n=20). Table 2 shows some of the indications broken down further to give an idea of some more specific indications for CS cited. Table 3 shows overall data by group of caesarean indication and a comparison between the two groups from October 2014 and February 2015. It also shows whether the decision for caesarean section made at that time appeared appropriate according to the author. This was a subjective decision made by the author. It should be noted that the author had greater clinical experience and qualifications than those making the original decision in almost all cases (96% of caesarean decision were made by intern doctors or midwives, 4% by one of the other senior doctors). Table 4 shows an alternative management that could have been performed safely that might have avoided a caesarean section, or where a caesarean section would have been done at a more appropriate time. There were 109 cases included that were considered not appropriate in the above analysis.

**Table 1:** Demographic data

Demographics	Total	Oct-14	Feb-15
Mean age	23.9 years	23.5 years	24.2 years
Primiparous (n=)	78	40	38
Multiparous (n=)	122	60	62
No previous CS (n=)	129	63	66
1 previous CS (n=)	38	20	18
2+ previous CS (n=)	33	17	16
Median gestation	39 weeks	39 weeks	39 weeks

**Table 2:** Caesarean section indication by group

Indication for CS	Number (%)
**Dystocia**	**88 (44%)**
**Fetal distress**	**37(18.5%)**
Meconium liquor	7
FH abnormality	12
Prelabour ROM	5
Oligohydramnios	5
HIV not on treatment	2
Cord prolapse	4
Genital warts	1
Polyhydramnios	1
**Increased risk of rupture**	**34 (17%)**
**Malpresentation**	**21(10.5%)**
oblique lie	2
Breech	9
Transverse lie	7
Compound presentation	2
Mento-posterior face	1
**Maternal/fetal compromise**	**20 (10%)**
Antepartum haemorrhage	9
Possible uterine rupture	6
Pre-eclampsia	3
Mother mentally disabled	1
“Precious baby”	1
**Total**	**200**

**Table 3:** Appropriateness of caesarean section

	Oct-14		Feb-15			Total	
Indication for CS	Number	Appropriate	Number	Appropriate	P-value	Number	Appropriate
**Dystocia**	45	11	43	14	0.48	88	25 (28.4%)
**Fetal distress**	16	4	21	7	0.72	37	11 (29.7%)
**Malpresentation**	9	4	12	6	1.0	21	10 (47.6%)
**Maternal/fetal compromise**	14	11	6	5	1.0	20	16 (80.0%)
**Increased risk of rupture**	16	13	18	16	0.65	34	29 (85.3%)
**Total**	**100**	**43**	**100**	**48**	**0.57**	**200**	**91 (45.5%)**

**Table 4:** Possible alternative management of inappropriate caesarean sections, n=109

Alternative management	Number (%)
Conservative management (e.g. allow more time for labour)	61 (60.0%)
Induction of labour	9 (8.3%)
Augmentation of labour (artificial rupture of membranes or oxytocin use)	28 (25.7%)
Instrumental (vacuum) delivery	11 (10.1%)

## Discussion

In the context of a unit with a known high CS rate, the principle findings of the study were of a disproportionately high number being performed for dystocia (44%). Alongside this was the findings that the decision for CS was appropriate in less than half of the cases (45.5%), and that this was even higher for cases performed for dystocia, where only 28.4% were appropriate. A cohort of interventions over the 4 months of the study period to try and reduce the number of unnecessary CS was unsuccessful with no difference in appropriateness found (p=0.57). The fact that only 4% of CS decisions were made by senior doctors is also an important finding. Finally, despite more complex interventions being available (e.g. induction/augmentation of labour and vacuum delivery) in a majority of cases (60.0%) conservative management would have reduced the number of unnecessary CS. During the literature search conducted, no studies were found looking at appropriateness of decision for CS. A strength of this study was that it attempted to address this issue, and link it with the indication for CS and explain why CS rates are high. Another strength was the attempted intervention and analysis to see if any change in appropriate CS could be achieved. Weaknesses of the study include the fact it was a retrospect analysis, so no true causality can be established despite the aims of the study. No power calculations were performed and it is likely that the number of cases was not sufficient to show a significant impact from the intervention between the two groups. The study was a single-centre study, so how applicable it is to other units or settings is questionable. Finally, the decision as to whether a CS was appropriate was subject, performed by one individual, making reproduction of the study difficult. The most common CS indication group by far was dystocia. This is in keeping with other studies from sub-Saharan Africa [5–7] and resource-rich settings [9]. However, all of these studies saw much lower proportions being performed for dystocia, 28-36% compared to the 44% we found. In a descriptive study of this nature no firm conclusions can be drawn as to the cause of this. However, the additional data collected showing only 28.8% of these were appropriate supports the notion that this proportion is too high because of unnecessary intervention. This theory can be further supported by the data collected on alternative managements, where a majority of cases could have been managed conservatively to try and allow more time for a vaginal delivery. Anecdotally reviewing the case notes a number of caesarean sections were being performed due to a suspected ‘big baby’ pre-labour or early in labour, often with intact membranes without a trial of labour in primiparous women. Also, a lack of appropriate alternative interventions to performing a CS was seen, with induction/augmentation of labour or vacuum delivery rarely performed.

Fetal indications were also higher than in other studies, but again this was a group was a very low percentage of appropriate CS being performed (29.7%). Again this theoretical correlation fits, in that unnecessary CS are being performed for this reason, pushing up the proportion performed for this reason, and pushing up the overall CS rate. Whilst fetal monitoring is difficult in a healthcare setting where only intermittent fetal auscultation is performed, many of these CS appeared unnecessary. Indications included just one episode of lower fetal heart rate, meconium in the absence of any other signs of fetal distress or concerning features, and ‘oligohydramnios’ in patients with known pre-labour ruptured membranes. For the majority of other CS indications the clinical picture is often clearer and involved a simpler clinical decision. For example, a patient who has had two or more previous CS has a significant increased risk of uterine rupture and a CS is a valid indication. Similarly, significant antepartum haemorrhage that might be a placental abruption presents immediate threat of life to mother and/or baby and again, is a justifiable CS. The final area where unnecessary CS were performed was in breech presentation (in the malpresentation group). The practice of CS for breech has spread throughout resource-poor settings without good clinical evidence [11] and this was seen in this study. Reasons why unnecessary CS were being performed that might explain the high CS rate are complex and beyond the scope of this study. Certainly clinical knowledge and experience might be thought to contribute, as seen by some of the example indications above. The interventions administered during the 4 months between the two groups unfortunately had no impact on the number of unnecessary CS, despite attempting to address this issue. There might be a number of reasons for this, including the effectiveness of these interventions; the amount of time between the two groups; and the ingrained clinical methods and unwillingness to learn or change behaviour amongst staff. Other factors that might contribute include resources and staff numbers. Certainly practice in resource-rich settings sees much greater numbers of clinician to patient ratios and often sees more clinical care, and the decision for CS, made by more senior doctors. For example in the UK every decision for CS must at least be discussed with a consultant [12], whereas a senior doctor only made the decision for CS in 4% in this study.

Overall the problem is so large it needs a fundamental change in the healthcare system in terms of resources, education, continuing professional development and clinical governance. There was no significant difference found following the educational interventions implemented by the author. The reasons for this cannot be established from the study and are likely to be multifactorial. As mentioned, the study may not have been powered appropriately to show any change, and even then there are no previous studies to base a power calculation on in terms of what sort of change in CS appropriateness would be expected. Other potential reasons for a lack of effect from the interventions include the suitability of the interventions. For example, staff and not used to using guidelines to base their practise on. Many staff could not attend the educational programmes implemented and the teaching sessions on how change practice based on new guidelines. New intern doctors rotate every few months so this will impact on practise. There are cultural issues with a UK based volunteer coming into a Uganda government hospital and implementing changes and whether this is appropriate. Also teaching methods may have been inappropriate or ineffective (for a number of reasons including the ability of the author, cultural differences in learning methods, etc.) Many staff have been practising according to certain methods for years and to impact this is likely to be difficult, and there maybe an unwillingness amongst some staff to take on new ideas. Finally, the impact of the intervention was only allowed 4 months to see if it had an effect, and an impact may have been apparent after a longer time period.

## Conclusion

Broadly speaking the indications for CS and their proportions are already known, but this study does add that in an individual unit those proportions may alter, as shown by the higher proportion of CS due to dystocia and fetal distress. In addition, this study also adds some background to this, by attempting to assess how many of these CS are appropriate, and showing that a majority were not appropriate. An attempt to reduce the proportion of inappropriate CS with a series of educational interventions over 4 months was unsuccessful. Reasons for these issues might include clinical knowledge and education, clinical resources (including staff numbers), and lack of senior clinician input. However, as a retrospective observational study no causality can be made. Further studies on the appropriateness of CS, and the impact of potential educational interventions to reduce unnecessary CS and reduce the CS rate are needed.

### What is known about this topic

- That CS rates in many tertiary centres in sub-Saharan Africa are likely to be too high, well above recommended levels from the WHO, even accounting for the complex caseload and referrals they receive;
- CS indications tend to broadly similar throughout the world, with dystocia being the largest group;
- The model of care in resource-rich countries is of significant senior involvement in the decision to perform a CS.

### What this study adds

- That in an individual unit dystocia and fetal distress can form an even higher proportion of caesarean sections, and this is likely related to caesarean sections being performed without an appropriate indication;
- That in resource-poor settings senior doctors are not being involved in the decision to perform a CS;
- That a short (4-month), broadly educational set of interventions by one individual is not sufficient to reduce the proportion of inappropriate caesarean sections.
